# Low-elevation conifers in California’s Sierra Nevada are out of equilibrium with climate

**DOI:** 10.1093/pnasnexus/pgad004

**Published:** 2023-02-28

**Authors:** Avery P Hill, Connor J Nolan, Kyle S Hemes, Trevor W Cambron, Christopher B Field

**Affiliations:** Department of Biology, Stanford University, Stanford, CA, USA; Woods Institute for the Environment, Stanford University, Stanford, CA, USA; Woods Institute for the Environment, Stanford University, Stanford, CA, USA; Department of Earth System Science, Stanford University, Stanford, CA, USA; Department of Biology, Stanford University, Stanford, CA, USA; Woods Institute for the Environment, Stanford University, Stanford, CA, USA; Department of Earth System Science, Stanford University, Stanford, CA, USA

**Keywords:** ecology, habitat suitability modeling, vegetation transitions, vegetation climate mismatch, climate change, California

## Abstract

Since the 1930s, California’s Sierra Nevada has warmed by an average of 1.2${}^{\circ }$C. Warming directly primes forests for easier wildfire ignition, but the change in climate also affects vegetation species composition. Different types of vegetation support unique fire regimes with distinct probabilities of catastrophic wildfire, and anticipating vegetation transitions is an important but undervalued component of long-term wildfire management and adaptation. Vegetation transitions are more likely where the climate has become unsuitable but the species composition remains static. This vegetation climate mismatch (VCM) can result in vegetation conversions, particularly after a disturbance like wildfire. Here we produce estimates of VCM within conifer-dominated forests in the Sierra Nevada. Observations from the 1930s Wieslander Survey provide a foundation for characterizing the historical relationship between Sierra Nevada vegetation and climate before the onset of recent, rapid climate change. Based on comparing the historical climatic niche to the modern distribution of conifers and climate, ∼19.5% of modern Sierra Nevada coniferous forests are experiencing VCM, 95% of which is below an elevation of 2356 m. We found that these VCM estimates carry empirical consequences: likelihood of type-conversion increased by 9.2% for every 10% decrease in habitat suitability. Maps of Sierra Nevada VCM can help guide long-term land management decisions by distinguishing areas likely to transition from those expected to remain stable in the near future. This can help direct limited resources to their most effective uses—whether it be protecting land or managing vegetation transitions—in the effort to maintain biodiversity, ecosystem services, and public health in the Sierra Nevada.

**Significance Statement**

Warming climatic conditions over the last century have led to observable shifts in the spatial organization of dominant tree species in California’s Sierra Nevada. Little is known, however, about the extent to which these shifts have tracked the magnitude of climate change. This study maps Vegetation Climate Mismatch in the Sierra Nevada—areas where climate change has left trees in climatic conditions where they have not historically occurred. Different vegetation types support different wildfire regimes, ecosystems, and ecosystem services. Our maps will be useful for anticipating vegetation transitions and informing long-term wildfire and ecosystem management across the Sierra Nevada mountains of California.

## Introduction

Warmer and drier conditions prime forests for ignition (1), but climate change also directly affects the species composition of future vegetation. Climate-driven vegetation conversion is an understudied phenomenon in general and a potentially significant determinant of catastrophic wildfire risk that could require changes in management strategies (2–4).

Broadly, climate change has caused vegetation to shift poleward and up-slope (5–7). In long-lived ecosystems like forests, climate change is occurring faster than the ability of many plants to shift their distributions or adapt, resulting in vegetation disequilibrium (8) or vegetation climate mismatch (VCM). Forests experiencing VCM are at risk of converting to alternative species assemblages, particularly after stand-replacing disturbances such as severe wildfire (4). In some cases, VCM can even make forests more susceptible to wildfires (9).

**Fig. 1.**
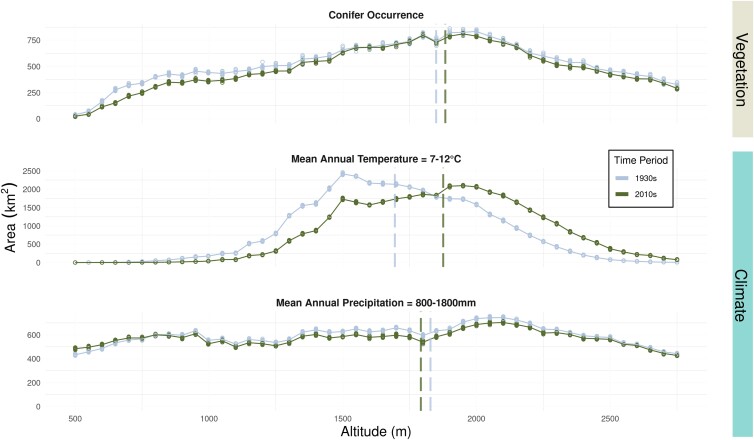
Observed elevation shifts in temperature, precipitation, and conifers across the study area between 80 years. The elevation distribution of modern conifers (top panel, dark green; mean = 1,884 m, SD = 640 m) was 34 m higher (95% CI = [25 m, 43 m]) than the 1930s conifers (top panel, light blue). This average shift in elevation was far less (by 145 m; 95% CI = [135 m, 156 m]) than the up-slope shift in the nominal 7–12${}^{\circ }$C Mean Annual Temperature envelope of Sierra Nevada low-elevation conifers (182 m; 95% CI = [179 m, 187 m]) (10). Note that the Mean Temperature envelope of Sierra Nevada low-elevation conifers would be shifted approximately 1${}^{\circ }$C cooler, if calculated based on the vegetation distribution in the Wieslander survey. Mean Annual Precipitation (bottom panel) decreased between the two time periods at most elevations—more-so at higher elevations—and the average elevation of the MAP envelope of Sierra low-elevation conifers decreased by 37 m (95% CI = [27 m, 49 m]). Circles represent samples with measurement error randomly introduced, and solid lines represent the averages across 10 samples. Vertical dashed lines show the total mean for the time period.

VCM is likely to be found in California’s transition zone between low-elevation conifer-dominated forest and angiosperm-dominated vegetation (including mixed chaparral, oak woodland, and mixed broadleaf forest) (Fig. 1; elevation ∼1000–1400 m)—where the foothills of the Sierra Nevada end and the mountains of the western flank begin. These forests lie on the warm end of mixed conifer distributions, where canopy dominants include ponderosa pine, sugar pine, and Douglas-fir (10), and understories are typically composed of mixed chaparral’s characteristic scrub oak, chaparral oak, and manzanita.

Boundaries between conifer-dominated forest and nonconifer vegetation at the western slope of the Sierra Nevada that were established under a previous climate regime may now be out of equilibrium with the current climate, especially if established trees continue to persist, even as climate conditions become unsuitable for seedlings and saplings of the same species. In these settings, stand-replacing fire—which pushes forests back to seedling stages—can trigger a rapid transition from one vegetation type to another (4, 11, 12). These transitions can potentially lead to the local loss of species, ecosystem services, and irrecoverable carbon stocks, depending on the vegetation that replaces these forests—and can also impact future risk of catastrophic wildfire.

Recent human population growth and large wildfires in these lower-elevation conifer forests punctuate the need to assess ecological stability, particularly as it relates to wildfire risk.

## Results and discussion

Conceptually, present-day VCM in Sierra Nevada coniferous forests exists if the geographic shift of tree species does not keep pace with climate change. This mismatch between vegetation and climate will make regeneration after disturbance more difficult. The Wieslander survey from the 1930s provides an anachronistically high resolution (minimum mapping unit of 16 ha) and expansive (176,900 km^2^) assessment of historical California vegetation (13, 14). Comparing the historical vegetation distribution to modern EVeg maps (15, 16), the mean elevation of conifers has shifted up-slope by 34 m on average (95% CI = [25 m, 43 m]). Over the same time period, the characteristic temperature range for conifers (10) has shifted up-slope by 182 m (95% CI = [179 m, 187 m]), based on historical and contemporary temperature and precipitation at 30 arc-second resolution (17) used to calculate 19 bioclimatic variables for 1915–1955 and 2000–2020. The magnitude of this temperature shift is approximately three to five times greater than the shift of the conifers (*Altitude*_*MAT*_ − *Altitude*_*conifer*_ = 145 m; 95% CI = [135 m, 156 m]), suggesting the presence of VCM (Fig. 1). In calculating the altitude shifts of both conifer occurrences and temperature variables, 10 pseudo-replicate sets were made by randomly introducing known measurement error (see methods) and bootstrapped 1000 times.

**Fig. 2.**
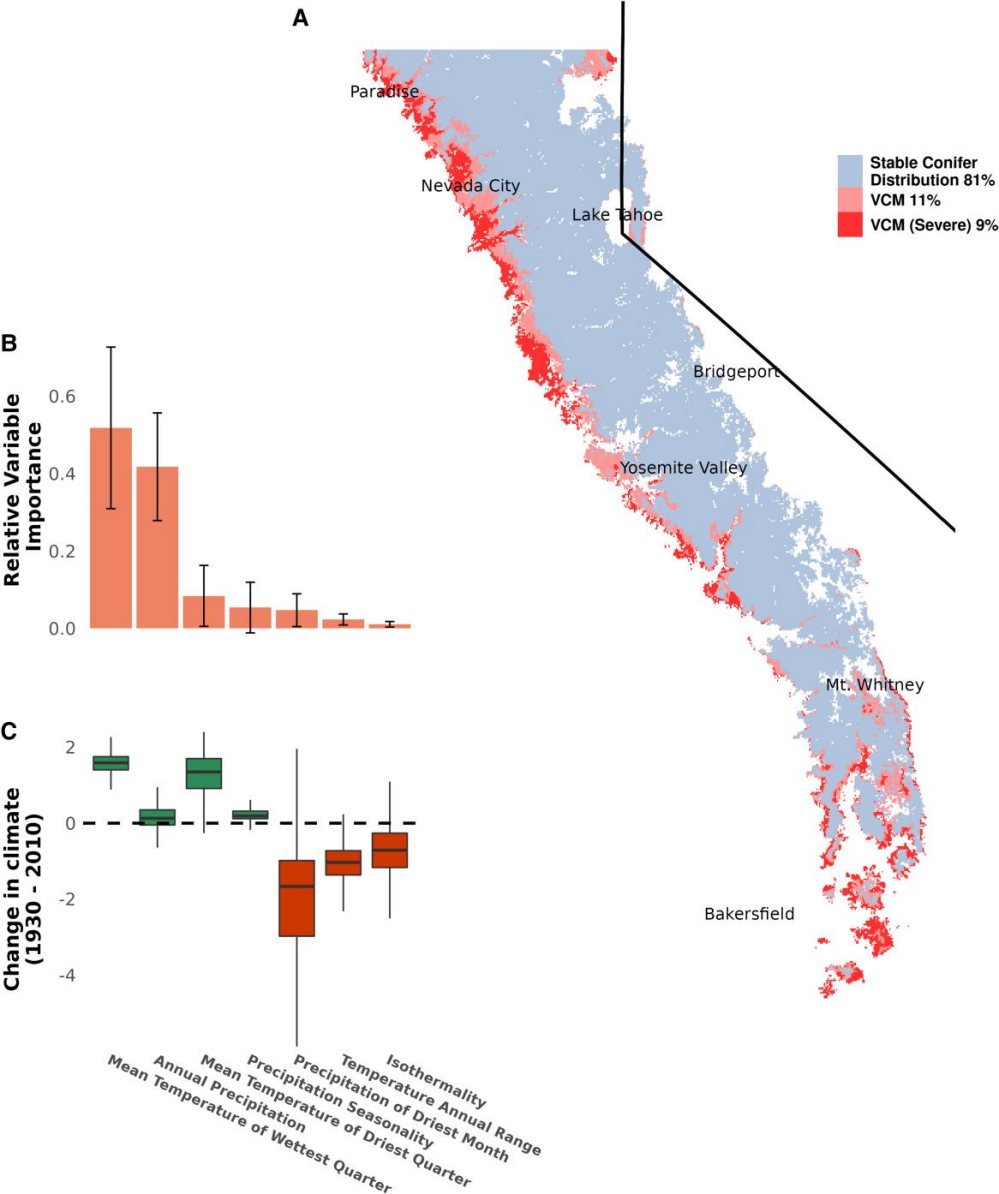
Estimated conifer VCM in the Sierra Nevada (2015–2020). (a) The conifer HSM projected to contemporary climate and overlayed on the modern conifer distribution (EVeg) reveals that up to 19.5% of modern conifer forest is in VCM, primarily along the low-elevation western slope of the Sierras. The total area of conifers shown is 40,495 km^2^, of which ∼32,500 km^2^ are in equilibrium with the modern climate. (b) Mean Temperature of Wettest Quarter and Mean Annual Precipitation were the most important predictors in the HSM (mean_MTWQ_ = 0.518, SE_MTWQ_ = 0.209 and mean_MAP_ = 0.418, SE_MAP_ = 0.139). Standard error bars are included in the barplot. (c) Boxplots show the difference between modern (2015–2020) and historical (1915–1955) climate within the conifer VCM regions. Change in climate is calculated as the number of standard deviations the modern climate differs from the historical period. Though the differences were statistically significant for each climate variable (*p* < 8.45 × 10^−12^, independent *t*-test), Precipitation of Driest Month showed the greatest decrease (mean = −2.41, SD = 2.84) and MTWQ the greatest increase (mean = 1.59, SD = 0.329) between the historical and modern climate. Mean Annual Precipitation changed the least within the VCM area (mean = 0.165, SD = 0.395). Boxplots include the median line, a box denoting the interquartile range, and whiskers showing values ±1.5 × the interquartile range.

To provide a more accurate and geographically explicit assessment of VCM, we quantified the climatic drivers of conifer distribution (i.e. the climatic niche) within the Sierra Nevada, using the 1930s vegetation data and 800 m resolution climate data (1915–1955) to train a habitat suitability model (HSM) for Sierran conifer forests. The advantage of using these older data is that they come from an era when the vegetation and climate were closer to equilibrium, before the vast majority of human-caused warming (18). Using the *sdm* (v 1.0-89) (19) and *dismo* (v 1.3-3) (20) packages in R (v 4.1.1) (21), we trained a Generalized Additive Model (GAM) on 56,844 conifer presence and 26,504 conifer absence points and 7 bioclimatic variables, using 5-fold spatial-blocking cross-validation for model evaluation (*AUC*_*test*_ = 0.94 ± 0.039, *COR* = 0.78 ± 0.079, *AUC*_*train*_ − *AUC*_*test*_ = 0.027 ± 0.042). Mean Temperature of the Wettest Quarter (MTWQ) and Mean Annual Precipitation (MAP) were the strongest determinants of conifer distribution in the 1930s, with 52% (SD=20.9%) and 42% (SD=13.9%) relative variable importance, respectively (Fig. 2b).

**Fig. 3.**
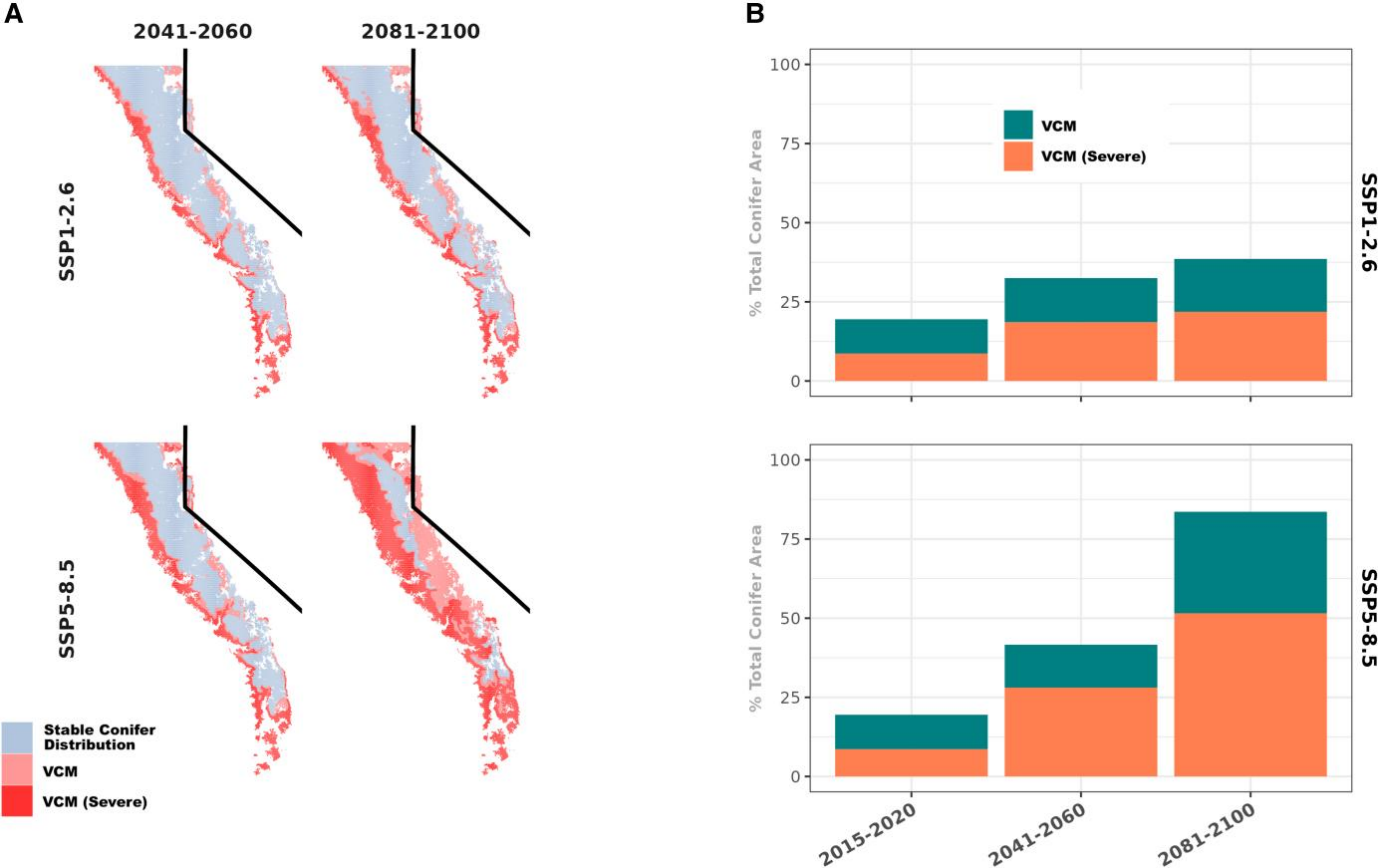
Future projections of VCM for two emissions pathways. Projection of the conifer HSM to mid-century and late-century climates suggest a dramatic increase in VCM by the end of the century if the contemporary conifer range edges do not move. The expected growth in severe VCM area (SSP1-2.6: 2.53 km^2^/yr; SSP5-8.5: 5.98 km^2^/yr) outpaced the more moderate VCM growth (SSP1-2.6: 1.54 km^2^/yr; SSP5-8.5: 2.95 km^2^/yr) under both emissions scenarios. Following the trend in projections of historical VCM (Fig. S1), VCM is expected to increase most along the western slope of the Sierra Nevada.

We used this model to predict regions of suitable conifer habitat across time periods from 1960 to 2100. CMIP6 data from scenarios SSP1-2.6 (an ambitious mitigation future) and SSP5-8.5 (a continued high emissions future) were used to predict future changes in habitat suitability. Habitat suitability (*HS*) was divided into three categories: suitable (*HS* ≥ 0.52), unsuitable (0.52 > *HS* ≥ 0.18), and severely unsuitable (0.18 > *HS*). These were determined based on habitat suitability thresholds above which 95% and 99% of the historical (Wieslander) conifers occurred (i.e. where sensitivity = 95% and 99%). In other words, less than 5% of historical conifer occurrences were in environmental conditions with *HS* < 0.52, which we characterize as “unsuitable” habitat. When compared with contemporary (2010s) EVeg maps of conifer distributions, these habitat suitability estimates reveal large, contiguous patches of conifer VCM in the Sierra Nevada—particularly along the low-elevation western slopes—that account for 19.5% of modern conifer forests (∼7,500 km^2^) (Fig. 2a). From 1960 to 2020, the area of conifer VCM has increased consistently (Fig. S1). When projected across the remainder of the 21st century, even the lowest emissions pathway (SSP1-2.6) leads to VCM doubling by the end of the century, if conifer range edges stay static (Fig. 3).

Based on the model, this increase in VCM is primarily attributable to an increase in Mean Temperature of the Warmest Quarter (MTWQ) across the study area between the 1930s and present day (Fig. 2c). The variable response curves (Fig. S2) demonstrate the sensitivity of conifer habitat suitability to high values of MTWQ: above a MTWQ of 0.5${}^{\circ }$C, habitat suitability drops by approximately 0.1 for every 1${}^{\circ }$C of warming. This sensitivity of low-elevation conifers to higher temperatures presumably reflects a suite of physiological features that make them less competitive against angiosperms under warm conditions. A number of nonclimatic environmental features, like edaphic characteristics and disturbance regimes, can also be important drivers of conifer range limits, but there is no evidence for consistent patterns of these along the conifer/angiosperm boundaries in the Sierra Nevada (10).

**Fig. 4.**
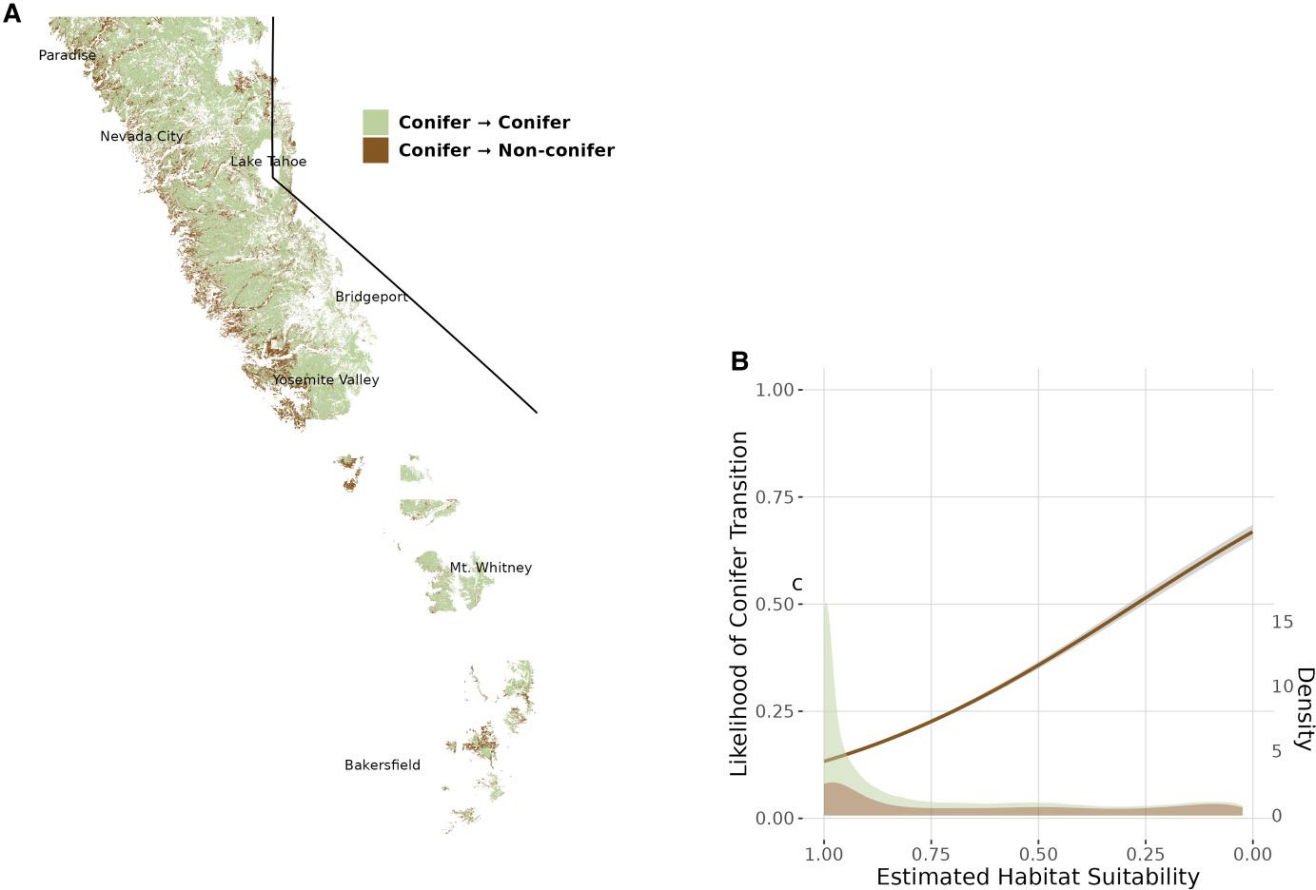
Habitat suitability of observed vegetation transitions between 1930s and 2010s. Areas that transitioned from conifer-dominated to angiosperm-dominated vegetation from 1930s to present tended to have lower modern conifer habitat suitability (*p* < 2 × 10^−16^). (a) All areas in the Sierra Nevada with complete vegetation data from the 1930s (Wieslander) and 2010s (EVeg) area shown. Most transitions from conifer-dominant vegetation are along the low-elevation edges of the historic conifer distribution. (b) The fitted logistic regression line indicates that the odds of conifer forests persisting decreased by 9.2% (95% CI = [0.092, 0.093]) for every 0.1 decrease in predicted habitat suitability. Probability density estimates for the areas of either transition or persistence are included.

Areas that transitioned from conifer-dominated in the 1930s to angiosperm-dominated in the 2010s generally have lower contemporary conifer habitat suitability than areas that maintained conifer dominance over that time period. Logistic regression indicates that the odds of conifer forests persisting decreased by 9.2% (95% CI = [0.092, 0.093]) for every 0.1 decrease in predicted habitat suitability (Fig. 4b). The areas of the Sierras where conifer-dominated vegetation transitioned to angiosperm-dominated vegetation occur primarily along the lower-elevation western slopes (Fig. 4a). This finding affirms the empirical implications of the low habitat suitability predicted by our model—these areas are at greater risk of eventually converting to nonconifer dominated vegetation. While it is difficult to tease apart the relative contribution of different possible drivers of vegetation transitions observed in this area, it is likely that decreased climatic suitability has compounded the impacts of other activities like logging or fire suppression.

These results are consistent with recent literature documenting observed and expected vegetation change in the Sierra Nevada and climate-induced vegetation transitions more broadly. A number of studies document observed or expected geographic shifts in low-elevation conifer-dominated forests, due at least in part to warming temperatures. Examples come from the Alps (22), Rocky Mountains (11), the state of California (23), and the Sierra Nevada (24). In the Sierra Nevada, studies using the Wieslander survey show that an increase in oak or hardwood vegetation is concomitant with a reduction in pine species prevalence (in western El Dorado county in particular, ponderosa pine decreased in area by 570 km^2^, and montane hardwood increased by 498 km^2^) (25, 26). Fire suppression has played a notable role in shaping the distribution and demography of these Sierra Nevada forests as well, and has worked in concert with warming temperatures to favor hardwood species like *Quercus spp.* while reducing the dominance of less shade-tolerant species like *Pinus spp.* (27). Conifer regeneration failure is likely a major driver of these observed and expected patterns, and researchers such as Shive et al. have found that a combination of climatic shifts and disturbance characteristics (e.g. burn severity) significantly affect the likelihood of conifer regeneration (28).

Is it possible that this analysis based on vegetation maps from the 1930s and the 2010s artificially inflates the area of VCM? Effects of logging prior to the 1930s and differences in mapping criteria warrant evaluation. The contrast between the projected and actual distribution of Sierran conifer forest depends on the robustness of the assumption that 1930s vegetation was in equilibrium with the climate. It is clear that there was little warming before the 1930s and that most of the anthropogenic climate change expected to cause VCM has been within the last few decades (29). Is it also possible that, even in the 1930s, vegetation was out of equilibrium with climate as a result of logging limiting conifers at the warm, dry end of their distribution? Logging was widespread throughout the Sierras in the late 19th and early 20th centuries. By 1945, ∼55% of nonsubalpine Sierra Nevada forests were second or third growth forest (30). We do not find evidence that logging consistently led to permanent vegetation conversions, and logged Sierran forests often regrow with the prelogging dominants. Ponderosa pine forests, for example, can return to dominance within 50 years of being clear-cut (31); Sierran mixed conifer, as few as 12 (32). We think it more unlikely that logging and other anthropogenic impacts would have specifically impacted the low-elevation edge of conifer distributions across the entirety of the Sierras in a way that would meaningfully truncate the estimated climatic niche. In the Manual of Field Instructions (33), Wieslander et al. write: “Except for the following, do not attempt to map smaller units than 40 acres of any type. (1) Remnant woodland and timber types in chaparral areas, or timber types in woodland would always be mapped if 10 acres or more” (pp. 39–40). The use of the word “remnant” implies that the surveyors were sensitive to vegetation transitions and made an effort to classify conifer types even after disturbance, which further mitigates the possibility that anthropogenic vegetation conversions prior to 1930 affected conifer range edges.

Even so, we cannot eliminate the possibility that pre-1930s logging or anthropogenic disturbance led to vegetation conversions and some overestimation of modern VCM. We explored the sensitivity of our results to the possibility that 1930s conifers were missing from the lower-elevation range edge as a result of human activity and, therefore, shifted toward the cool end of their climatic niche (opposite the pattern that has emerged since the 1930s). To do this, we trained HSMs on Wieslander data manipulated such that the highest elevation nonconifer samples were randomly converted to conifer samples. Our results followed the expectation that the area of modern VCM decreases with an assumed expansion of lower-elevation historical conifer distribution (Fig. S6). However, even under the extreme scenario in which the Wieslander survey did not detect conifer vegetation over 2,700 km^2^ of the lowest elevation regions that may once have contained conifers, total VCM is still >10% of all modern conifer forests within the study area.

Vegetation mapping criteria were generally similar for the maps from the 1930s and the 2010s, but there are subtle differences in vegetation classification and minimum mapping unit (MMU) size (25). Under the CALVEG classification system, an area is classified as the taller of a set of possible vegetation types if the taller type (e.g. coniferous) occupies >10% of the mapping unit. In contrast, Wieslander VTM has a threshold of 20%. Because the EVeg maps lean towards classifying areas with only a few conifers as conifer-dominant vegetation, the modern maps might exaggerate conifer area at low elevations, contributing to an overestimate of VCM. Likewise, the difference in MMU between the two maps may contribute to an overestimate of modern conifer VCM. The MMU of EVeg (≤1 ha) is smaller than that of the Wieslander data (16 ha total; 4 ha for “timber types” (33)), so EVeg data are more likely to register smaller stands of conifers, which may be more common along the lower-elevation edge of conifer distributions. To compensate for this, our vegetation aggregation method for both the 1930s and 2010s maps is intentionally sensitive to conifer occurrences, registering conifer presence if ≥5% of an 800 m (64 ha) grid cell contains conifer vegetation.

Our first-of-a-kind maps of areas experiencing VCM represent a new consideration when planning for forest management. These VCM forests are at risk of failing to regenerate after a disturbance. The exact mechanisms and sequences of events that lead to vegetation change will vary across the landscape and should be a target of future studies. But overall, VCM requires a move away from simply resisting fire and vegetation change to a more active management approach that directs the changes in a way that is beneficial to ecosystems and the nearby communities (34, 3).

Incorporating VCM into forest management plans will require experimentation and a delicate balancing of constituencies and their interests. There will likely be tradeoffs to be negotiated and difficult decisions to be made. For example, the public is often reticent to engage in large scale thinning or prescribed burns due to economic and esthetic reasons. But these very interventions may be important for forest health and fire safety. These and other interventions that move away from traditional resilience and towards a more “adaptive” or “transformative” resilience are likely to be necessary (35).

Understanding a region’s habitat suitability also influences management choice after a disturbance event. Most notably, in a VCM area, efforts to reforest after a fire or other disturbance with the same vegetation type as before are unlikely to be successful. Post-disturbance restoration needs to take into account the species mix and density that can currently be supported, but also the kinds of vegetation that future conditions are likely to support. This requires managers grappling with uncertainty in climate projections as they plan the future of the lands they manage; accessible tools that can synthesize climate projections with species distribution modeling can facilitate this planning process.

Maps of vegetation-climate mismatch can also inform conservation priorities. Habitats that are in equilibrium today and projected to remain in climate equilibrium should be prioritized for protection. In contrast, habitats that are out of equilibrium today or that are projected to go out of equilibrium could be treated to reduce the risk of catastrophic fires. Alternatively, transitions to new vegetation types could be facilitated in forests experiencing VCM. Schemes that incentivize ecosystem management for climate mitigation, like California’s forest offset program (36), will need to integrate nonstationary future climate and vegetation risks and opportunities (37).

## Conclusion

We have identified, quantified, and mapped vegetation climate mismatch in the Sierra Nevada: a new risk factor relevant to long-term management of catastrophic wildfire. Up to 19.5% of conifer forests are in areas that no longer have suitable climate for conifer regeneration. Thus, when there is a disturbance, such as a large fire, the conifer forest will be unlikely to reestablish. Conifer forests experiencing VCM may also be out-competed by vegetation types like mixed broad-leaf forests and chaparral that are better suited to the new climate and often grow more quickly than conifers, especially at the seedling stage.

Impending vegetation shifts across such a significant portion of California require a change in management strategy and a more long-term framing of catastrophic fire-risk in California.

Tools to prioritize treatment and protection are desperately needed given that more than 20% of California’s forestland would benefit from fuel treatments; myriad barriers, including funding, stand in the way (38). Our maps of conifer forest VCM provide new guidance on what types of management are likely to be successful and where. Investments made in better prioritizing conservation, fuel management, and fire mitigation in high-risk forestlands can have compounding returns—economically, ecologically, and in the form of human health and well-being.

## Materials and methods

### Vegetation data

The US Forest Service’s Wieslander Survey (1928–1940) provides the oldest spatially explicit, landscape-scale vegetation data in California. The surveyors mapped dominant vegetation (at a minimum mapping unit of 16 ha) through a combination of plot surveys and remote observation from peaks and vistas (13). We accessed the digitized and georeferenced shapefiles of these maps through Berkeley’s Vegetation Type Mapping Project Collection (vtm.berkeley.edu), which cover an area of more than 175,000 km^2^ across California (14). While digitizing the maps, Kelly et al. translated the vegetation classification system used by Wieslander to the more contemporary and widely used California Wildlife Habitat Relationships models (CWHR) (39). The geographic error of the basemaps ranges between 127 and 462 m (mean = 232 m) (40).

The Wieslander survey also collected plot data, with ostensibly higher spatial and taxonomic resolution. We used the Wieslander vegetation maps, rather than the plot data, for 4 reasons. (1) The vegetation data provide a much larger sample size than the plot data when up-scaled to 800 m resolution (Fig. S5). Besides the plot data clearly being more sparse, there are also large tracts of land that are mapped in VTM for which there are no plots available (e.g. Lake Tahoe Basin). (2) The more contiguous vegetation data allowed us to estimate the percent cover of different vegetation types and more effectively reconcile the difference in resolution between the occurrence and climate data used to train the habitat suitability model (further details below). (3) The Wieslander vegetation maps appear to be the Wieslander survey’s primary data product, and the plots are more of an exercise in surveyor training/ground-truthing (33). From the Wieslander Manual of Field Instructions: “The plots serve as a check on the mapper’s field judgment and assist him in an understanding of types. They are used immediately in the field for this purpose” (p. 74). (4) The digitized Wieslander plot data do not include CWHR vegetation data, only species lists, and the primary goal of this study is to find patterns at the scale of vegetation.

We sourced recent vegetation data from the US Forest Service’s EVeg (Existing Vegetation) maps for the North Sierra and South Sierra regions (15, 16). These vector maps were produced from source data including NAIP and WorldView-2 using the CALVEG (Classification and Assessment with Landsat of Visible Ecological Groupings) classification system, and have a horizontal positioning accuracy of ∼50 m. The North Sierra data were produced from satellite images from 2000 to 2014, while the South Sierra source data ranged from 1995 to 2016. The CALVEG vegetation classes “crosswalk easily” to CWHR classes, which are provided in the EVeg map product (41).

We cropped all vegetation data to the general extent of the Sierra Nevada mountains, which we derived from the Northwestern Forested Mountains ecoregion (42) east of the Central Valley and south of 40${}^{\circ }$. We added a 45 arc-minute (∼70 km) buffer to the southern, western, and eastern extents to include vegetation data from surrounding lower-elevation areas, where available.

### Climate data

We sourced contemporary and historical monthly precipitation, maximum temperature, and minimum temperature data from Oregon State University’s PRISM (Parameter-elevation Regressions on Independent Slopes Model) Climate Group at 30 arc-second resolution (17). PRISM data are widely used and produced from the interpolation of observations from a multitude of US meteorological stations using a regression model which weights grid cells by their physiographic similarity to the station. Mean absolute error is 1${}^{\circ }$C for temperature variables and 10% for precipitation variables in the western US (43).

We used the biovars() function from the *dismo v. 1.3-3* R package to convert the monthly precipitation and temperature variables to 19 bioclimatic variables—including physiologically relevant variables such as mean diurnal temperature range and precipitation of the driest quarter—and produced averages for the following time periods: 1915–1955, 1960–1980, 1980–2000, 2000–2010, 2010–2020, 2015–2020.

The climate data for future scenarios came from the Coupled Model Intercomparison Project Phase 6 (CMIP6). We chose the Shared Socioeconomic Pathways (SSPs) SSP1-2.6 and SSP5-8.5 for the 2041–2060 and 2081–2100 time periods from the CanESM5 (Canadian Centre for Climate Modelling and Analysis, Canada) global circulation model. We chose CANESM5 because its projections for future climate in California are near the middle of results from CMIP6 (44). We downloaded a set of 19 bioclimatic variables at 2.5 arc-minute resolution from the Worldclim dataset (http://www.worldclim.org, accessed on April 27th, 2021) (45). SSP1-2.6 and SSP5-8.5 represent the lowest and highest potential emission scenarios for the next century, and are derived from estimates of future energy and land-use trajectories (46).

### Conifer habitat suitability model

We started building the Sierra Nevada conifer habitat suitability model by identifying all CWHR habitat types that were conifer-dominated within the study area. These included Sierran Mixed Conifer, Subalpine Conifer, Douglas Fir, Eastside Pine, Jeffrey Pine, Closed-Cone Pine-Cypress, Lodgepole Pine, Pinyon-Juniper, Ponderosa Pine, Red fir, and White fir. The Montane Hardwood-Conifer type is defined as at least 33% conifer-dominated and 33% hardwood-dominated vegetation (39), which we classified as 50% conifer presence and 50% conifer absence. All other explicitly nonanthropogenic CWHR types were considered conifer absences within the context of the model. To mediate the large difference in resolution between our occurrence and climate data we effectively up-scaled the binary occurrence data. We calculated the percent-cover of conifer presence and absence polygons within the 30 arc-second grid cells of the climate data. If 5% of the grid cell contained conifers, we considered it a presence. If the grid cell contained less than 5% conifer cover and nonconifer vegetation exceeded 5% then it was classified as an absence. Our threshold was chosen to reduce omission error so that the resulting habitat suitability model would capture the breadth of the climatic niche of conifers in the Sierra Nevada.

To reduce collinearity among the 19 bioclimatic predictors within the extent of our study area, we calculated the Variance Inflation Factor (VIF) for the set using the R package *usdm* (47) and incrementally excluded collinear variables until VIF < 10, as recommended. The 7 remaining variables were Mean Temperature of Wettest Quarter, Mean Annual Precipitation, Mean Temperature of Driest Quarter, Precipitation Seasonality, Precipitation of Driest Month, Temperature Annual Range, and Isothermality. Reducing collinearity among predictors helps to increase model efficiency and mitigate uncertainty (48). When transferring models across space or time, differences in predictor collinearity between training data and projecting data can lead to poor performance. We quantified collinearity shift by comparing the correlation matrices of historical predictors to those of the present and future (49). Among the 7 climate variables vetted for collinearity, the greatest absolute shift in *r* was 0.183 for Isothermality and Mean Annual Precipitation and the average absolute shift in *r* was 0.005 (Fig. S3).

All of our habitat suitability modeling was completed using the *sdm* (v 1.0-89) (19) and *dismo* (v 1.3-3) (20) packages in R (v 4.1.1) (21). We used five different presence-absence modeling algorithms available from the *sdm* package: Random Forest, Multivariate Adaptive Regression Spline, Generalized Linear Model, Boosted Regression Tree, and BIOCLIM with default settings. We trained each model on historical occurrence and predictor data and used 5-fold cross validation with spatial blocking (block size = 70 km, *blockCV* (v 2.1.4) package (50)) to partition the occurrence data into testing and training sets in order to evaluate model performance. *k*-fold cross validation works by splitting source data into *k* groups (or “folds”) and iteratively withholding each group as a “test” set while models train on the other *k* − 1 groups. The metrics of model performance are averaged across all (*k*) iterations. We used both AUC (the area under the receiver characteristic operating curve) and COR (point-biserial correlation coefficient) as model evaluation metrics. Because model extrapolation was a key feature of this work, we quantified the extent of over-fitting in the models by subtracting the training AUC from the test AUC (51). We selected the GAM model because GAM can perform better than other popular SDM methods when extrapolated to novel environments (52). Test statistics (Fig. S4A) and variable importance (Fig. S4B) across the different methods show that each method produced models with high AUC and COR and the most important predictors were MAP and MTWQ. The relative weighting of MAP and MTWQ was the greatest difference between the models, with GAM, MARS, and GLM weighting MTWQ relatively higher than the decision tree-based methods.

We calculated a series of thresholds for the historical conifer HSM to delineate the probability of presence into three categories: suitable habitat, unsuitable habitat, and severely unsuitable habitat. We defined these thresholds using model sensitivity (i.e. the proportion of true conifer occurrences that are classified as suitable conifer habitat at a given threshold of habitat suitability) where unsuitable habitat was defined as habitat suitability values under which 5% of all Wieslander conifer occurrences occurred (i.e. sensitivity = 0.95). Similarly, severely unsuitable habitat was defined by the habitat suitability threshold which excluded 1% of Wieslander occurrences (i.e. sensitivity = 0.99).

The HSM was used to predict conifer habitat suitability at seven different time slices throughout the 20th and 21st centuries, sourcing PRISM climate variables for present-day and historical time periods and CMIP6 for future projections. At every time slice, we intersected our HSM predictions with observed conifer occurrences from EVeg, to produce maps of conifer forests that grow in suitable, unsuitable, or severely unsuitable climates. We down-scaled the future projections to 30 arc-seconds via bilinear interpolation to match the resolution of the other time periods and simplify the comparison of relative area. Estimations of VCM are approximate for time periods outside of the present-day because EVeg conifer maps only reflect the modern distribution.

## Supplementary Material

pgad004_Supplementary_Data

## Data Availability

Wieslander survey data are available thanks to the efforts of the VTM Digitization Project (vtm.berkeley.edu). Contemporary vegetation data are available from US Forest Service (www.fs.usda.gov). Historical and contemporary climate data at 800 m resolution are available from the PRISM Climate Group (prism.oregonstate.edu). Future climate data are available from CMIP6 (esgf-node.llnl.gov/projects/cmip6/). Data and code for our analysis are available at github.com/avephill/sierra-nevada-VCM.
