# Supplementary material for: Low-elevation conifers in California’s Sierra Nevada are out of equilibrium with climate
Source: PNAS Nexus. 2023 Feb 28;2(2):pgad004. doi: 10.1093/pnasnexus/pgad004 (PMC9976749; doi:10.1093/pnasnexus/pgad004)
Supplement: pgad004_Supplementary_Data [file pgad004_supplementary_data.pdf]

## Supplementary materials

Figs. S1 to S6

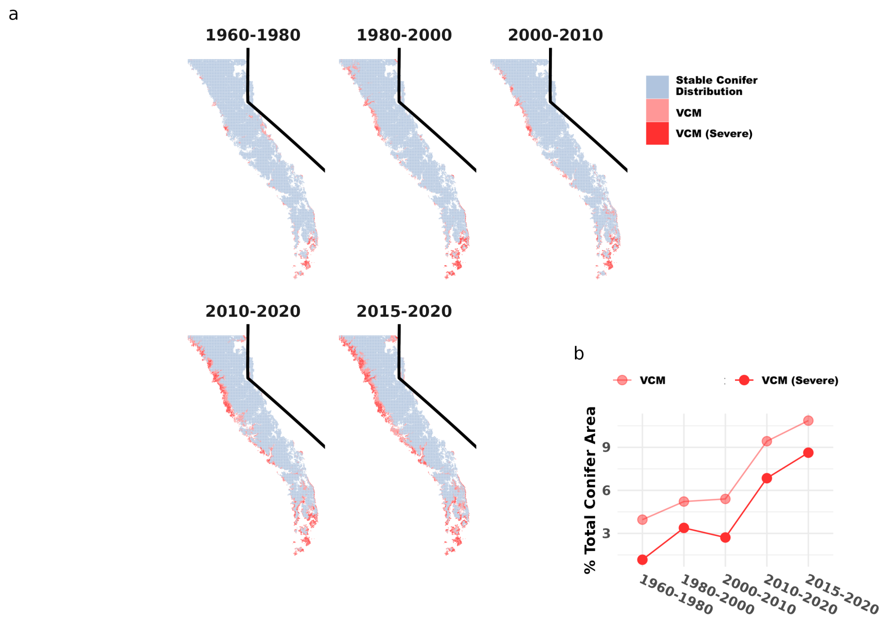


**Fig. S1. Historical time series of VCM.** This figure exhibits the decline in suitable conifer habitat from the 1960s to the present day. The conifer distribution shown is the modern EVeg distribution and provides a backdrop for illustrating the increase in unsuitable habitat as predicted by the HSM—the actual conifer range of the 20th century time periods is not represented by these data. Suitable habitat recedes along the western slope over time despite stalling briefly at the turn of the century. The modern 10-year climate average yields a slightly lower estimate of VCM than the 5-year average presented in Figure 2, but still predicts that 16.3% (6590km$^2$) of conifer forests are currently outside of climate equilibrium.

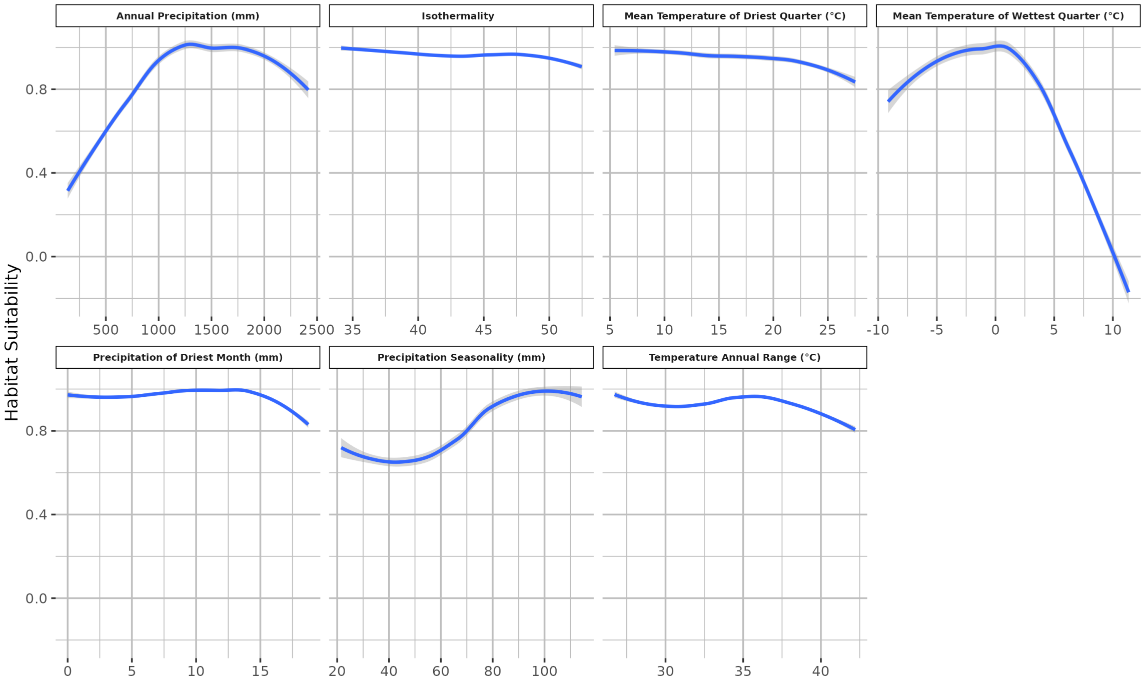


**Fig. S2. Predictor response curves of conifer HSM.** Both Mean Annual Precipitation and Mean Temperature of the Wettest Quarter were the two most important model predictors (Figure 2b and Figure S4b). Estimated conifer habitat suitability drops significantly at low values of MAP (below 1000mm) and high values of MTWQ (above 1°C). Results show the averages across the 5 folds and shaded areas indicate the 95% confidence interval.

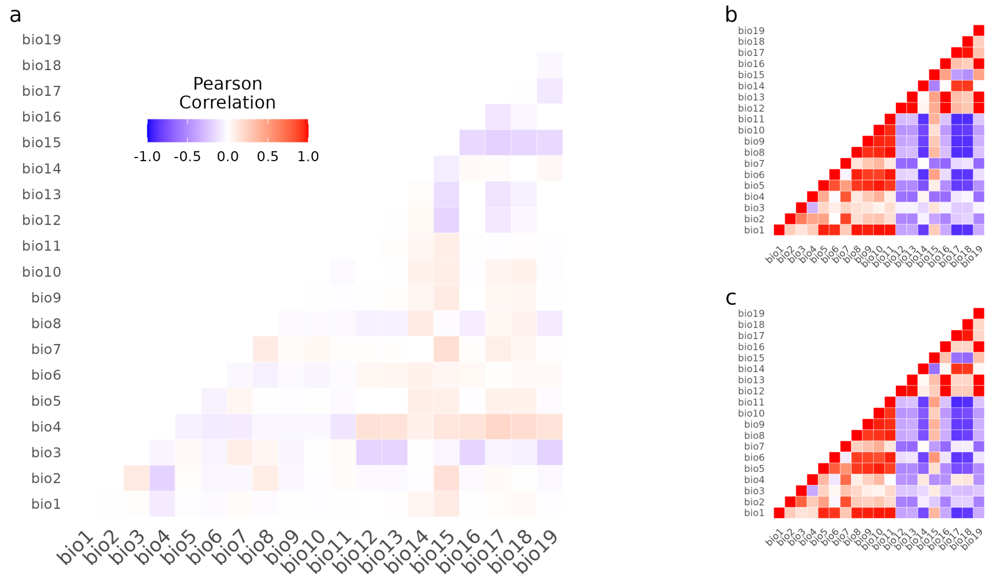


**Fig. S3. Collinearity shift between 2015-2020 climate and 1915-1955 climate averages for all 19 bioclimatic variables.** (a) The collinearity shift matrix is the difference between the 1915-1955 matrix (b) and the 2015-2020 collinearity matrix (c). The greatest collinearity shift occurred with bio4 and bio17 (Temperature Seasonality and Precipitation of Driest Quarter, respectively). Bio13 (Precipitation of Wettest Month) and bio19 (Precipitation of Coldest Quarter) had the smallest shift. Bio16 (Precipitation of Wettest Quarter) and bio19 (Precipitation of Coldest Quarter) had the greatest collinearity across both historical and contemporary time periods. Only bio3, bio7, bio8, bio9, bio12, bio14, bio15 were used to train the conifer HSM.

**a**

| Method | AUCtest | COR | Deviance | AUCtrain | AUCtest - AUCtrain |
|---|---|---|---|---|---|
| RF | 0.953 ± 0.016 | 0.809 ± 0.033 | 0.508 ± 0.087 | 1.000 ± 0.000 | -0.047 ± 0.016 |
| BRT | 0.946 ± 0.017 | 0.786 ± 0.048 | 0.720 ± 0.077 | 0.954 ± 0.003 | -0.008 ± 0.020 |
| GAM | 0.941 ± 0.024 | 0.794 ± 0.036 | 0.593 ± 0.164 | 0.964 ± 0.003 | -0.024 ± 0.025 |
| GLM | 0.938 ± 0.025 | 0.760 ± 0.045 | 0.614 ± 0.123 | 0.950 ± 0.003 | -0.011 ± 0.026 |
| MARS | 0.932 ± 0.029 | 0.748 ± 0.048 | 0.654 ± 0.162 | 0.956 ± 0.004 | -0.024 ± 0.031 |

**b**

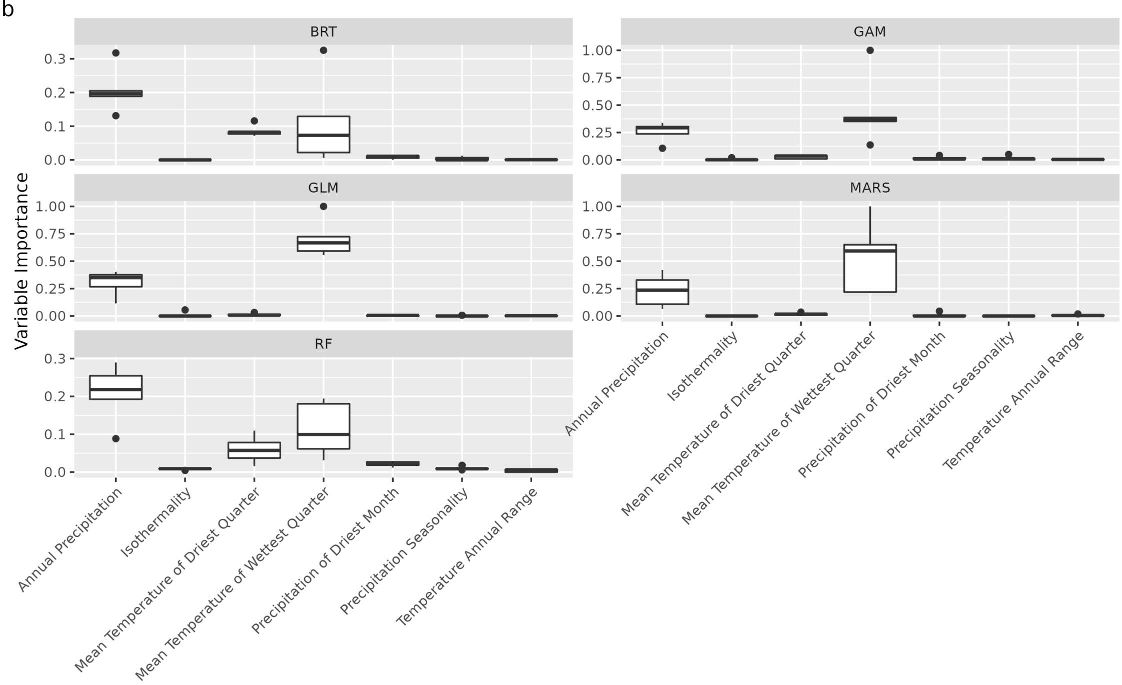


**Fig. S4. Model performance and predictor importance for models produced by alternative HSM methods.** We used GAM as the primary HSM method, but the results from different modeling methods show generally consistent results. All models were trained on the same data using 5-fold spatial blocking cross validation. (a) Random forest had the highest test AUC but also the greatest difference between test AUC and training AUC (which suggests some measure of overfitting). MARS had the lowest test AUC and COR. (b) All methods identified MAP and MTWQ as the most important predictors, but while GAM, GLM, and MARS ranked MTWQ as the most important, the decision tree-based methods (RF and BRT) ranked MAP higher.

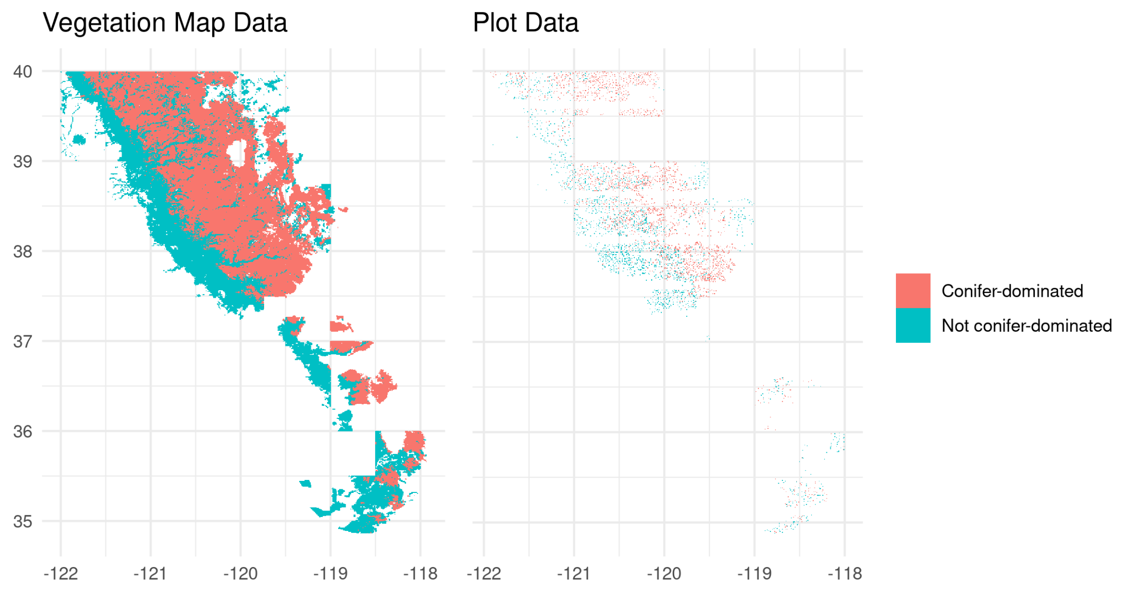


**Fig. S5. Comparison of Wieslander data sources after up-scaling to 800m resolution.** We prepared 2 sources of Wieslander vegetation data for HSM by aggregating them to the spatial resolution of the PRISM predictor variables (800m). The Vegetation Type Mapping (VTM) data source yielded a far larger sample size of conifer occurrence data (N = 76,467) than the plot data (N = 4,251) for use in training the historical conifer HSM. This is largely due to the vegetation maps being spatially contiguous while the plot data are more sparse, and further, that the vegetation map product was a more central focus of the Wieslander team ([51] pg. 49, 76). While aggregating, the plot data were classified as containing conifer-dominated vegetation if conifer dominants (according to CALVEG types) were present.

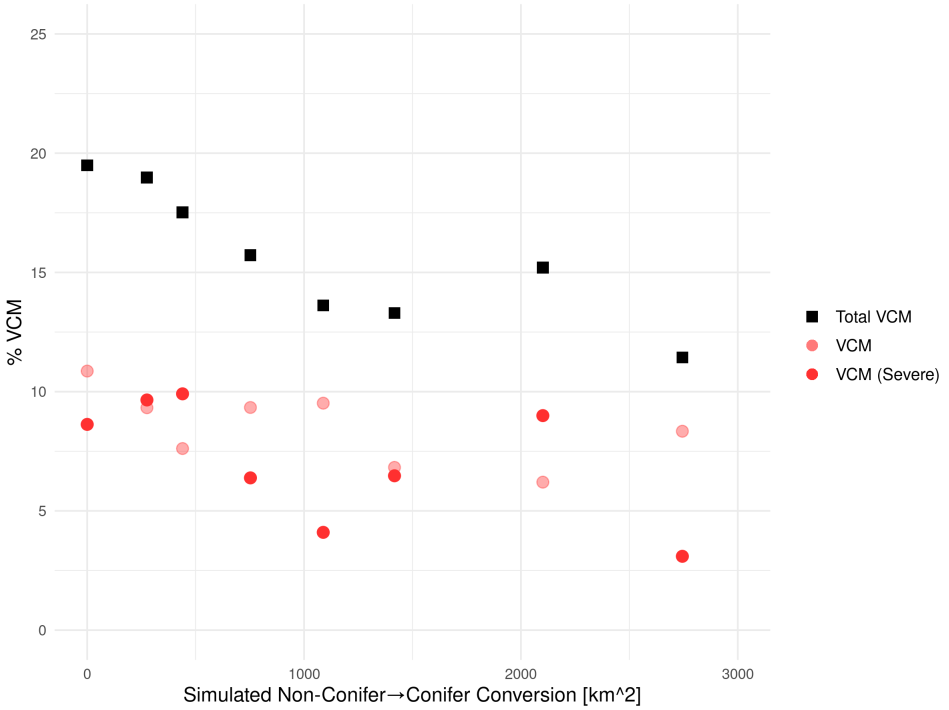


**Fig. S6. The simulated impact of low-elevation, anthropogenic vegetation transitions on estimated VCM.** We evaluated the sensitivity of our VCM estimates to potential anthropogenic type-conversion prior to the 1930s. Our method for calculating modern conifer VCM would overestimate it if anthropogenic activity (logging, changes to fire regime) caused conifer-dominated vegetation to convert to non conifer-dominated vegetation at the lower-elevation edge of the conifer distribution before the Wieslander survey was conducted. Therefore, we calculated modern VCM using simulations of Wieslander vegetation data with increasing expansions of the lower-elevation edge of conifer-dominated vegetation. These are effectively a set of estimations of the counterfactual where conifer-dominated vegetation was not converted to non conifer vegetation prior to the Wieslander survey. We randomly converted 2.5%, 5%, 10%, 15%, 20%, 30%, and 40% (expressed in $km^2$ on the x-axis) of grid cells within the highest elevation 50% of non-conifer grid cells along the western slope of the Sierras to conifer grid cells. Resulting VCM calculations follow the expectation that modern VCM decreases with a lower-elevation historical conifer distribution (and therefore, broader observed climatic niche). However, even under the unlikely scenario in which the Wieslander survey did not detect conifer vegetation within $2700km^2$ of the lowest elevation regions that once potentially contained conifers (a staggering 9% of the area of all regions within the study area where Wieslander detected conifer vegetation), our modern estimation of total VCM is still greater than 10% of all modern conifer forests.
